# *Leishmania *isoenzyme polymorphisms in Ecuador: Relationships with geographic distribution and clinical presentation

**DOI:** 10.1186/1471-2334-6-139

**Published:** 2006-09-13

**Authors:** Manuel Calvopina, Rodrigo X Armijos, Jorge D Marco, Hiroshi Uezato, Hirotomo Kato, Eduardo A Gomez, Masataka Korenaga, Paola A Barroso, Tatsuyuki Mimori, Philip J Cooper, Shigeo Nonaka, Yoshihisa Hashiguchi

**Affiliations:** 1 Department of Parasitology, Kochi Medical School, Kochi University, Kochi 783–8505, Japan; 2 Health Sciences Program, College of Health Sciences, University of Texas at El Paso, El Paso, TX 79902–0581, USA; 3 Department of Dermatology, Faculty of Medicine, University of the Ryukyus, Okinawa 903–0215, Japan; 4 Department of Veterinary Hygiene, Faculty of Agriculture, Yamaguchi University, Yamaguchi 753–8515, Japan; 5 Departamento de Medicina Tropical, Facultad de Medicina, Universidad Católica Santiago de Guayaquil, Ecuador; 6 Department of Microbiology, School of Health Sciences, Kumamoto University, Kumamoto 862–0976, Japan; 7 Laboratorio de Investigaciones, Hospital Pedro Vicente Maldonado, Pichincha Province, Ecuador; and Centre for Infection, St George's University of London, London, UK

## Abstract

**Background:**

Determinants of the clinical presentation of the leishmaniases are poorly understood but *Leishmania *species and strain differences are important. To examine the relationship between clinical presentation, species and isoenzyme polymorphisms, 56 *Leishmania *isolates from distinct presentations of American tegumentary leishmaniasis (ATL) from Ecuador were analyzed.

**Methods:**

Isolates were characterized by multilocus enzyme electrophoresis for polymorphisms of 11 isoenzymes. Patients were infected in four different ecologic regions: highland and lowland jungle of the Pacific coast, Amazonian lowlands and Andean highlands.

**Results:**

Six *Leishmania *species constituting 21 zymodemes were identified: *L. *(*Viannia*) *panamensis *(21 isolates, 7 zymodemes), *L. *(*V.*) *guyanensis *(7 isolates, 4 zymodemes), *L. *(*V.*) *braziliensis *(5 isolates, 3 zymodemes), *L. *(*Leishmania*) *mexicana *(11 isolates, 4 zymodemes), *L. *(*L.*) *amazonensis *(10 isolates, 2 zymodemes) and *L. *(*L.*) *major *(2 isolates, 1 zymodeme). *L. panamensis *was the species most frequently identified in the Pacific region and was associated with several clinical variants of cutaneous disease (CL); eight cases of leishmaniasis recidiva cutis (LRC) found in the Pacific highlands were associated with 3 zymodemes of this species. Mucocutaneous leishmaniasis found only in the Amazonian focus was associated with 3 zymodemes of *L. braziliensis*. The papular variant of CL, Uta, found in the Andean highlands was related predominantly with a single zymodeme of *L. mexicana*.

**Conclusion:**

Our data show a high degree of phenotypic variation within species, and some evidence for associations between specific variants of ATL (i.e. Uta and LRC) and specific *Leishmania *zymodemes. This study further defines the geographic distribution of *Leishmania *species and clinical variants of ATL in Ecuador.

## Background

Ecuador straddles the Andes and is crossed by the Equator and is home to extremely diverse ecologic conditions. American tegumentary leishmaniasis (ATL) in Ecuador occurs throughout the tropical Amazon and Pacific coastal regions and in some inter-Andean valleys. According to the Ecuadorian Ministry of Health, human cases have been reported from 20 of the country's 22 provinces and an estimated 3.1 to 4.5 million people are considered to be at risk of infection with 3,000–4,500 new cases occurring annually [1].

Differences in clinical presentations and severity of the leishmaniases are explained by interactions between particular molecular and biologic characteristics of different *Leishmania *species, host genetics and immunity and, environmental factors [2,3]. Although it is generally accepted that major clinical forms of ATL may be caused by specific *Leishmania *species, the relationship between *Leishmania *zymodemes and clinical presentation remains unclear [4]. To date, most studies investigating the relationship between polymorphisms and clinical disease have provided little evidence for strong associations [5-9], probably because of the substantial variability among the etiological agents of ATL at the subgenus level, with at least 15 species described [10]. In addition, *Leishmania *species in the New World are highly diverse genetically as reported from Brazil, Colombia, French Guiana and Peru [7-9,11,12].

ATL in Ecuador constitutes a group of diseases with a wide spectrum of clinical variations [13,14] and atypical presentations [15]. Six *Leishmania *species had been identified as causative agents of ATL in this country: *L. *(*Viannia*) *braziliensis*, *L. *(*V.*) *panamensis*, *L. *(*V.*) *guyanensis*, *L. *(*Leishmania*) *mexicana*, *L. *(*L.*) *amazonensis *and *L. *(*L.*) *major*-like [13,16-19]. However, few parasite isolates have been analyzed for genetic diversity or phenotypic characterization by isoenzyme polymorphisms and a survey of 28 human stocks isolated from single and multiple CL ulcers showed a high degree of genetic diversity within *L. panamensis*/*L. guyanensis *species [20].

The characterization and identification of causative species and zymodeme phenotypes, and their geographic distribution together with the clinical disease caused provides important information for the design, prioritization (e.g. according to risk of mucosal disease), and implementation of control programs for this important disfiguring parasitic infection. In the current study, we report the diversity of *Leishmania *species and zymodeme phenotypes isolated from clinical lesions from 56 patients with varying clinical presentations of ATL from 4 different geographic regions of Ecuador.

## Methods

### Origin, sampling, isolation and cultivation of *Leishmania *stocks

Twenty-nine parasite isolates included in this study were sampled between 2000 and 2001 in the Leishmaniasis Reference Center of the Central University, and the remaining isolates were obtained during an active search for patients infected in the Pacific highlands and lowlands, Amazonian lowlands and, in the Andean region. None of the patients had been living or infected outside Ecuador.

**Figure 1:**
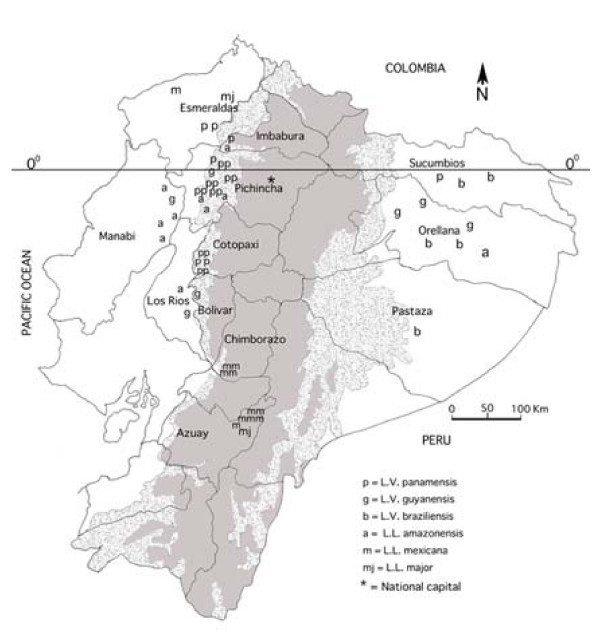
**Map of Ecuador showing the geographic locations (regions and provinces) of *Leishmania *species identified in this study**. The shaded area shows the Andean plateau (>1,000 m altitude). Dotted areas show highland jungle or Andean slopes (400–1,000 m elevation). The tropical rainforest of Pacific coastal and Amazon regions are shown in white. Each letter represents one isolate.

Ecologically, (i) the highlands (500–1,000 m altitude) and (ii) lowlands (<500 m) of the Pacific region are subtropical and tropical humid rainforest, respectively. Both zones are predominantly forest cleared for cattle ranching or cultivation of banana, cocoa, coffee, and African palm oil; the ambient temperature ranges 15–22°C in the highlands and 24–28°C in the lowlands. (iii) Amazonian lowlands (100–500 m elevation) are located East of the Andes and are covered by dense tropical rainforest with temperatures varying 25–29°C and high humidity. (iv) inter-Andean valleys (Huigra and Paute) are at 1,200–2,500 m elevation with temperatures ranging from 12–18°C and scarce vegetation (Figure 1).

*Leishmania *isolates were obtained by aspiration of lesions from patients with suspected CL or MCL and aspirates were cultivated directly in USMARU biphasic medium as described previously [21]. The parasites were bulk-cultured in RPMI-1640 medium with 20% fetal calf serum. They were harvested by centrifugation (10,000 *g *× 10 min) and washed thrice in phosphate-buffered saline (pH 7.2) as described [22]. Parasites pellets were stored at -80°C until isoenzyme analysis. All patients provided written consent to participate in the study and the protocol was approved by the ethical committee of the Central University of Quito, Ecuador.

### Isoenzyme characterization

Sample preparation, electrophoresis and staining procedures were followed as described [22,23]. Cellulose acetate plates (Sebiagel, Moulineaux, France) were used and assays were repeated twice. In this study, we have assayed each Ecuadorian isolate and WHO reference strain for the activity of the following 11 enzymatic systems: malate dehydrogenase (EC 1.1.1.37, MDH); malic enzyme (EC 1.1.1.40, ME); 6-phosphogluconate dehydrogenase (EC 1.1.1.44, 6-PGD); glucose-6-phosphate dehydrogenase (EC 1.1.1.49, G-6-PD); mannose phosphate isomerase (EC 5.3.1.8, MPI); glucose phosphate isomerase (EC 5.3.1.9, GPI); alanine aminotransferase (EC 2.6.1.2, ALAT); aspartate aminotransferase (EC 2.6.1.1, ASAT); phosphoglucomutase (EC 2.7.5.1, PGM); pyruvate kinase (EC 2.7.1.40, PK) and nucleoside hydrolase (inosine) (EC 2.4.2, NHi). Nine selected WHO reference strains, obtained from the cryobank of London School of Hygiene and Tropical Medicine, were used in this study for species identification: *L. *(*V.*) *braziliensis *(MHOM/BR/75/M2903); *L. *(*V.*) *panamensis *(MHOM/PA/71/LS94); *L. *(*V.*) *guyanensis*. (MHOM/BR/75/M4147); *L. *(*V.*) *peruviana *(MHOM/PE/84/LC39); *L. *(*V.*) *naiffi *(MDAS/BR/79/M5533); *L. *(*L.*) *mexicana *(MNYC/BZ/62/M379); *L. *(*L.*) *amazonensis *(MHOM/BR/73/M2269); *L. *(*L.*) *chagasi *(MHOM/BR/74/M2682) and, *L. *(*L.*) *major *(MHOM/SU/73/5ASKH).

### Data analysis

Each isolate was assessed according to the position of the electrophoretic bands for all 11 enzymes. Each electrophoretic band was considered as a separate character and was numbered from the most distal to the anodic point in each zymogram. Zymodemes were identified according to the pattern of electrophoretic profiles for the 11 enzymes as previously described [24]. Identification of the Ecuadorian isolates was performed by comparing electrophoretic profiles with WHO reference *Leishmania *strains. Hierarchical cluster analysis was used to summarize the relationships between zymodemes using Jaccard's coefficient of similarities [25]. Phenograms were constructed by analyzing each electrophoretic band as a unit character, by construction of a matrix with the presence or absence of electrophoretic bands using the interactive molecular evolutionary genetics analysis (MEGA) software, version 3.0 [26].

## Results

A total of fifty-six *Leishmania *isolates were obtained from fifty-six different subjects with ATL in Ecuador. Individuals were infected in four different bioclimatic geographic areas in 12 provinces (Figure 1). Of the 56 subjects studied, 52 presented with cutaneous lesions with a wide variety of clinical presentations: 30 ulcers, 9 small papules or Uta-like, 8 leishmaniasis recidiva cutis (LRC), 2 sporotrichoid or pian-bois, 1 disseminated (DL), 1 erysipeloid and 1 with diffuse cutaneous leishmaniasis (DCL). The remaining four presented with mucocutaneous leishmaniasis (MCL) or espundia.

**Figure 2:**
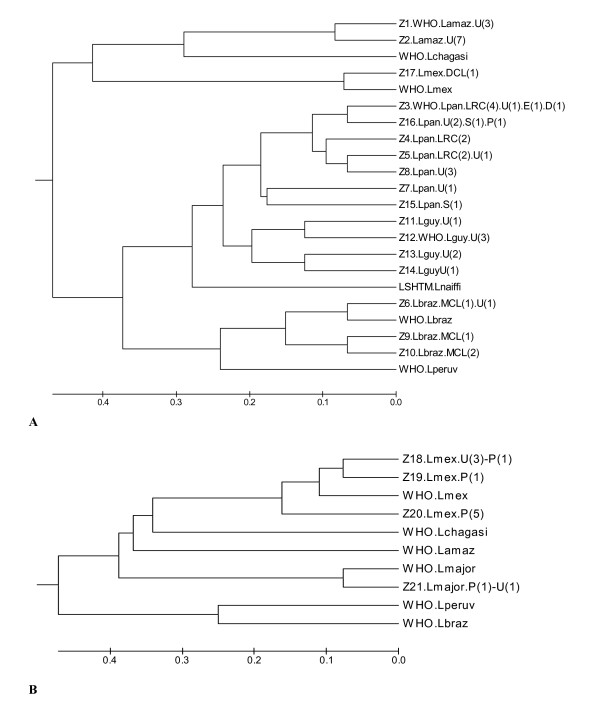
**Phenogram of the 56 Ecuadorian *Leishmania *isolates and 9 WHO reference strains**. 2A shows the 17 zymodemes from patients infected in the forest of Pacific and Amazon region. 2B shows 4 zymodemes identified from the inter-Andean area. The *Leishmania *species identified are shown at the end of the branches with the type of clinical lesions and number of subjects in parentheses. Lamaz – *L. amazonensis*, Lbraz – *L. braziliensis*, Lguy – *L. guyanensis*, Lmex – *L. mexicana*, Lpan – *L. panamensis*, Lperuv – *L. peruviana*. U, ulcer; DCL, diffuse cutaneous leishmaniasis; LRC, leishmaniasis recidiva cutis; E, erysipeloid; S, sporotrichoid; P, papular or Uta-like, and MCL, mucocutaneous leishmaniasis.

In total, six *Leishmania *species were identified revealing 21 different zymodemes (Table 1). Of the 56 isolates, 21 (37.5%) were typed as *L. panamensis *revealing 7 different zymodemes; 11 (19.6%) as *L. mexicana *with 4 zymodemes; 10 (17.8%) as *L. amazonensis *with 2 zymodemes; 7 (12.5%) as *L. guyanensis *with 4 zymodemes; 5 (8.9%) as *L. braziliensis *with 3 zymodemes and 2 (3.6%) as *L. major *with one zymodeme. The zymodeme composition is shown in Table 2. None of the *Leishmania *zymodemes present in Ecuador of *L. braziliensis*, *L. mexicana *and *L. major *were identical to the *Leishmania *WHO reference strains and, none of the Ecuadorian stocks were matched to the WHO reference strains for *L. *(*V.*) *peruviana*, *L. *(*V.*) *naiffi *or *L. *(*L.*) *chagasi *(Figure 2A and 2B).

**Table 1:** Distribution of *Leishmania *species and zymodemes by lesion type and geographic origin

		Geographical region			
		
*Leishmania *species	Zymodeme	Pacific lowlands	Pacific highlands	inter-Andean valleys	Amazonian lowlands
*L. L. amazonensis*	Z1	ulcer (1)	ulcer (1)	**-**	ulcer (1)
	Z2	ulcer (4)	ulcer (3)	**-**	**-**
*L. V. panamensis*	Z3	**-**	LRC (4), ulcer (1), E (1), DL (1)	-	**-**
	Z4	-	LRC (2)	-	-
	Z5	-	LRC (2), ulcer (1)	-	-
	Z7	ulcer (1)	-	**-**	-
	Z8	ulcer (1)	ulcer (1)	-	ulcer (1)
	Z15	-	S (1)	-	-
	Z16	-	ulcer (2), papular (1), S (1)	**-**	-
*L. V. guyanensis*	Z11	ulcer (1)	-	**-**	**-**
	Z12	-	-	**-**	ulcer (3)
	Z13	**-**	ulcer (2)	**-**	**-**
	Z14	**-**	ulcer (1)	**-**	**-**
*L. V. braziliensis*	Z6	-	-	-	ulcer (1), MCL (1)
	Z9	-	-	-	MCL (1)
	Z10	-	-	-	MCL (2)
*L. L. mexicana*	Z17	DCL(1)	-	-	-
	Z18	-	-	ulcer (3), papular (1)	-
	Z19	-	-	papular (1)	-
	Z20	-	-	papular (5)	-
*L. L. major*	Z21	ulcer (1)	-	papular (1)	-

Number of isolates are shown in parentheses. DCL, diffuse cutaneous leishmaniasis; LRC, leishmaniasis recidiva cutis; MCL, mucocutaneous leishmaniasis; S, sporotrichoid; E, erysipeloid.

**Table 2:** Enzymatic profiles of the 21 zymodemes found in *Leishmania *isolates from Ecuador

	Enzymes										
	
*Leishmania *spp. and zymodeme	G-6PD	6-PGD	ME	MDH	ASAT	ALAT	GPI	MPI	PGM	PK	NH
*L. L. amazonensis*											
Z1	2	1	1	2	5	2	3	2	4	2	2
Z2	2	1	1	2	4	2	3	2	4	2	2
*L. V. panamensis*											
Z3	2	4	2	1,5	1	3	1,2	4	3	1	1,3
Z4	2	4	2	1,5	2	3	1,2	4	3	1	1,3
Z5	2	4	3	1,5	1	3	1,2	4	3	1	1,3
Z7	1	3	3	1,5	2	3	1,2	4	3	1	1,3
Z8	2	4	3	1,5	2	3	1,2	4	3	1	1,3
Z15	1	4	3	1,5	1	3	1,2	4	2	1	1,3
Z16	2	2	2	1,5	1	3	1,2	4	3	1	1,3
*L. V. braziliensis*											
Z6	2	3	2	1,3	3	4	1,2	3	2	3	1,4
Z9	2	3	3	1,3	2	6	1,2	3	2	3	1,4
Z10	2	3	2	1,3	2	6	1,2	3	2	3	1,4
*L. V. guyanensis*											
Z11	1	2	3	1,5	2	3	1,2	4	4	1	2,3
Z12	1	2	3	1,5	2	5	1,2	4	3	1	2,3
Z13	1	2	3	1,5	1	4	1,2	4	3	1	2,3
Z14	2	4	3	1,5	1	4	1,2	4	3	1	2,3
*L. L. mexicana*											
Z17	3	1	1	1,3	4	1	4	1	1	3	2,4
Z18	3	2	3	2	5	1	4	3	2	3	4,6
Z19	3	2	3	2	5	1	4	3	1	3	4,6
Z20	4	2	2	3	5	1	4	3	2	3	4,6
*L. L. major*											
Z21	4	2	4	1	2	2	2	1	4	3	4,6

There was some evidence for geographic restriction of *Leishmania *species: *L. panamensis*, *L. guyanensis *and *L. amazonensis *were isolated from 3 regions (not from the inter-Andean); *L. braziliensis *was isolated only from the Amazon lowlands; *L. mexicana *was identified almost exclusively in the inter-Andean region, although one isolate of this species was identified in the Pacific lowlands (Figure 1).

**Figure 3:**
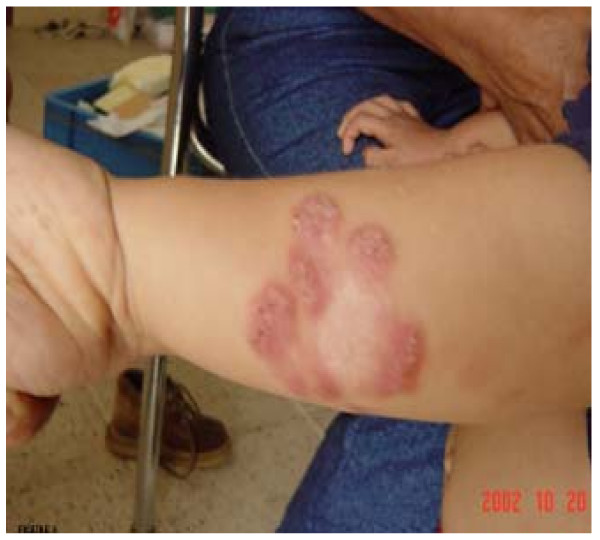
**Lesions of Leishmaniasis recidiva cutis (LRC) on the forearm of a 6-years old boy**. Red-brown papules appeared around a healed scar and progressed intermittently over a period of 3 years, leaving an extensive depressed scar (center). All parasites isolated from LRC were identified as *L. *(*V.*) *panamensis*, entailing 3 (Z3-Z5) out of 7 zymodemes.

Findings for each of the 56 isolates are provided in Table 1 showing *Leishmania *species and zymodeme identified for each of the 4 ecologic regions in Ecuador. In the Pacific coastal highlands, 25 human isolates were studied, 12 were obtained from cutaneous ulcers and 13 from atypical presentations (LRC, sporotrichoid, erysipeloid and DL); three species, *L. panamensis*, *L. guyanensis *and *L. amazonensis *and 10 different zymodemes were identified as causative organisms. Three zymodemes of *L. panamensis *(Z3, Z4 and Z5) were the only parasites associated with LRC (See Figure 3 for clinical characteristics and Figure 2A for zymodemes and their distance with WHO *L. panamensis*). Interestingly, Z3 was identified also in other atypical variants of CL as disseminated and erysipeloid. Ulcers were associated with 8 zymodemes from the 3 species circulating in Pacific highlands. In the Pacific lowlands, 10 isolates were studied, of which 9 came from single or multiple CL ulcers and 1 from a patient with DCL. Five species with 7 different zymodemes were identified from these isolates. The case of DCL was associated with Z17 of *L. mexicana*.

**Figure 4:**
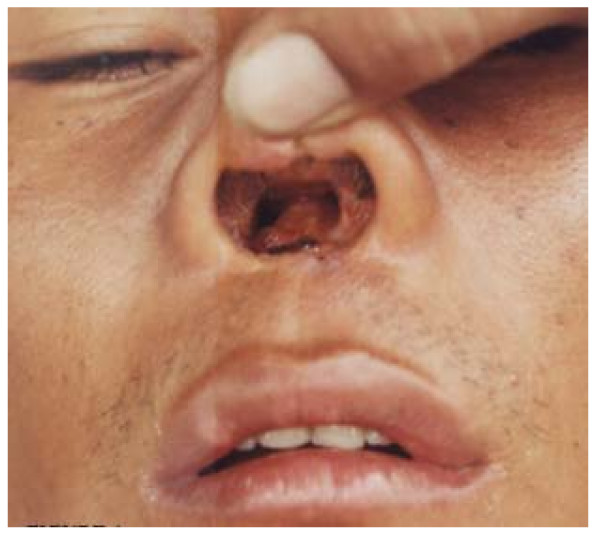
**Mucocutaneous leishmaniasis (MCL) or espundia**. Active lesions in nasal mucosal tissue with septal perforation and disfigurement of the nose and swelling of upper lip in a 38-years old man native from the Amazonian lowlands. Parasites from MCL cases were identified as *L*. (*V*.) *braziliensis *but no relationship with a specific zymodeme was observed.

**Figure 5:**
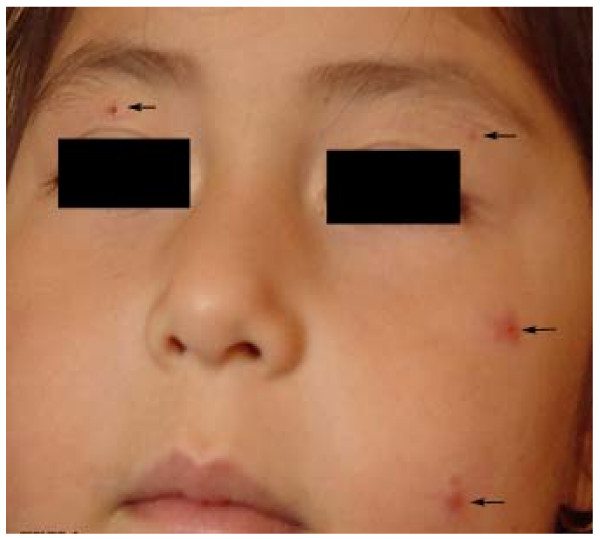
**Andean Leishmaniasis or Uta**. Four small painless lesions (arrows) in a girl of 5-years old living in the highlands of Ecuadorian Andes. Lesions generally appear on the face of children, and heal within 6 months. All parasites isolated from Uta lesions were *L*. (*L*.) *mexicana *with a predominance of zymodeme 20. No mucosal lesions were seen in these foci.

Ten isolates were analyzed from the Amazon lowlands of which four came from MCL (Figure 4) and 6 from CL ulcers. Four species and 6 zymodemes were identified. Three zymodemes of *L. braziliensis *(Z6, Z9 and Z10) were associated with the isolates from MCL patients. Eleven isolates were studied from the inter-Andean valley region, 7 isolates from Paute and 4 from Huigra, of which 10 were identified as 3 zymodemes (Z18, Z19 and Z20) of *L. mexicana *and one a single zymodeme of *L. major*. Most clinical lesions in this region were crusted papules (Uta-like) lesions (Figure 5).

The phenograms (Figure 2) show that the *Leishmania *subgenera, *Viannia *and *Leishmania*, form distinct groups. The genetic distance (Jaccard distances) between the two subgenera was over 0.9. Figure 2A shows the relationships between different zymodemes isolated from the subtropical and tropical Pacific and Amazon geographic regions, while Figure 2B shows the same for the inter-Andean zymodemes. Different species were considered have Jaccard distance values greater than 0.48. The 17 zymodemes, including WHO reference strains, within the *Viannia *group (lower branch of phenogram) could be divided into five main species clusters for *L. panamensis, L. guyanensis, L. naiffi, L. braziliensis*, and *L. peruviana*. The four species of *Leishmania *subgenus included in this study (*L. mexicana*, *L. amazonensi*s, *L. major*, and *L. chagasi*) revealed 10 zymodemes (upper branch of phenogram), 10 Ecuadorian isolates were identified as *L. amazonensis *and two isolates as *L. major *(Z21). From the eleven isolates identified as *L. mexicana *involving 4 zymodemes, Z20 was the only zymodeme identified in 5 out of 8 Uta lesions and confined to Paute. No isolates of *Viannia *subgenus were found in the inter-Andean region.

## Discussion

The data from this study shows a great diversity of *Leishmania *species and zymodeme phenotypes isolated from a wide spectrum of clinical forms of ATL in Ecuador. The diversity in the parasite populations may contribute to the highly variable clinical spectrum of leishmaniasis observed in this study and previous studies [13-16,27]. A high degree of enzymatic polymorphisms in *Leishmania *parasites has been reported previously in Ecuador [20], and also in the neighboring countries of Colombia, Brazil and Peru [8,9,12]. The findings of higher molecular diversity in species isolated from subtropical and tropical regions of the Pacific coast in this study rather than from the inter-Andean region could be related to the greater number of animal reservoirs and sandfly fauna encountered in these regions [28,29]. We report for the first time in Ecuador enzymatic polymorphism of *L. amazonensis*, *L. mexicana *and *L. braziliensis*, and have characterized *Leishmania *from mucocutaneous lesions.

The present study provides evidence that some of the distinct clinical forms of ATL are associated with specific *Leishmania *species and some may be related with a restricted number of zymodemes. Thus, MCL was associated only with *L. braziliensis*, LRC and other atypical presentations of CL with *L. panamensis *and, Uta lesions with *L. mexicana*. The evidence for associations of specific zymodemes with clinical variants was observed only for LRC and Uta; three zymodemes of *L. panamensis *associated with all 8 patients with LRC, and 1 zymodeme of *L. mexicana *related with 5/8 cases of Uta. It has been suggested that clades of *L. braziliensis *genotypes may be associated by specific cutaneous, mucosal or disseminated manifestations [9] but our data provides limited evidence for this and is in agreement with other studies [5-8,11]. *L. braziliensis *was the only species isolated from MCL lesions and previous studies in Brazil, Colombia and Peru have linked also mucosal involvement to infection with this species [8,9,30].

*L. panamensis *believed to cause generally cutaneous ulcers [31] in this study was associated with all lesions of LRC and other presentations (i.e., erysipeloid, sporotrichoid, and disseminated). However, the latter lesions were observed only in the Pacific highlands (Andes slopes) and it is possible that factors specific to this environment may contribute to atypical presentations of ATL such as volcanic soil [32].

*L. mexicana *infection presented in children as small papules (Uta) in the inter-Andean region, lesions that heal spontaneously in a few months leaving an imperceptible scar. *L. mexicana *isolated in the Andes seems to have a unique life cycle, in which only *Lu. ayacuchensis *and domestic dogs are incriminated as vector and reservoir, respectively [28,33]. Interestingly, in the Peruvian Andes, a similar clinical presentation is caused by *L. peruviana *[34]. It has been speculated that UV radiation, which is stronger in the highlands, may also play a role in the outcome of infection [35].

This study demonstrated geographic restriction of some *Leishmania *species in Ecuador. *Leishmania *subgenus was generally observed in the inter-Andean region, whereas *Viannia *was observed generally in forested areas. However, there was some overlap with *L. mexicana *and *L. major*, also being isolated from the Pacific region. *L. panamensis *was the predominant species identified in the Pacific highlands (76.0% of all isolates) and *L. mexicana *predominated in the inter-Andean region (90.9% of isolates). In general, the geographic distribution of *Leishmania *species observed in this study is in agreement with previous reports [16,18-20]. Nevertheless, we did not identify any isolates of *L. braziliensis *from the Pacific region, and this finding may explain the rarity of MCL in this region. Although mucosal involvement caused by this and others *Viannia *species has been reported in the Colombian Pacific coastal [8], we and others clinical investigators have never diagnosed espundia originating in the Pacific coastal area of the country.

Interestingly, *L. amazonensis *was identified in 17% of the 56 isolates examined. This figure is higher than previously reported but our data confirmed the wide distribution of this species in forested areas of the Pacific Ecuador [13,16,17,19] and we identified this species in the Amazon region. The original observation of *L. major*-like infection in Ecuador recorded by Hashiguchi et al., [18] has been confirmed in the present study. Furthermore, we have demonstrated that these parasites belong to different zymodeme of the WHO reference strain (Turkmen, USSR). *L. major *has been identified also in Brazil, Venezuela, Paraguay and Mexico [9,24,36]. It has been reported that *L. naiffi *is widespread in South American countries [37] with one patient possibly infected in Ecuador. In the current study we were unable to isolate any parasite with phenotypic similarity to the WHO reference stock for this species.

## Conclusion

We have identified six *Leishmania *species and 21 zymodemes associated with parasites isolated from 56 patients with different clinical forms of leishmaniasis from diverse ecological areas of Ecuador. Our data show a high degree of species and enzymatic polymorphism and some evidence for major clinical presentations of leishmaniasis to be associated with specific *Leishmania *species but limited evidence with zymodemes. Our data further defines the geographic distribution of leishmaniasis in Ecuador with respect to species distribution and the clinical presentations observed. The mapping of the geographic distribution of *Leishmania *species and clinical disease will be important both regionally and nationally for designing control programs or interventional field trials for drugs or vaccines.

## Competing interests

The author(s) declare that they have no competing interests.

## Authors' contributions

MC collected clinical samples, performed the isoenzyme electrophoresis, analyzed data and drafted the manuscript. RXA isolated and cultivated *Leishmania *parasites at the Central University. JDM, PAB and MK assisted with the isoenzyme electrophoresis. HU, HK, EAG, TM and SN isolated parasites in active field surveys. PJC analyzed data and helped write the manuscript. YH participated in the design of the study and sample collection.

## Pre-publication history

The pre-publication history for this paper can be accessed here:


